# Environmental exposures and pediatric kidney health

**DOI:** 10.1038/s41390-026-05174-2

**Published:** 2026-06-06

**Authors:** Anisha Gerber, Zoe Cacanindin, Julia Scialla, Nivedita Kamath

**Affiliations:** 1 Division of Pediatric Nephrology, Department of Pediatrics, University of Virginia School of Medicine, Charlottesville, VA USA; 2 School of Nursing, University of Virginia, Charlottesville, VA USA; 3 Division of Nephrology, Department of Medicine, University of Virginia School of Medicine, Charlottesville, VA USA; 4 Department of Public Health Sciences, University of Virginia School of Medicine, Charlottesville, VA USA; 5 Department of Pediatric Nephrology, St. John’s Medical College, St. John’s National Academy of Health Sciences, Bengaluru, India

## Abstract

**Abstract:**

Environmental exposures are increasingly associated with kidney and cardiometabolic disease in children. This review summarizes current understanding of the potentially hazardous effects of air pollution, chemical toxicants, and heavy metal contamination on pediatric kidney health and cardiometabolic risk factors for chronic kidney disease (CKD), as well as the potentially protective effect of green space. We highlight the critical windows of childhood development, during which the kidneys may have varying susceptibility to environmental exposures. We also discuss challenges and possible solutions to adequately powered studies at the intersection of environmental health and pediatric kidney disease. Environmental exposures are both ubiquitous and modifiable. Therefore, elucidating the contribution of environmental exposures to the increasing global burden of CKD will be crucial to informing strategies for kidney disease prevention, starting in childhood.

**Impact:**

- We summarize current evidence on the associations between environmental exposures—including air pollution, chemical toxicants, and heavy metal contamination—and pediatric kidney disease and related cardiometabolic risk factors, as well as the potentially protective effect of green space.
- We highlight how the impact of environmental exposures on the kidneys may vary during different critical windows of childhood development.
- Lastly, we discuss methodological challenges and potential solutions, such as using novel technologies and approaches to adequately power studies toward the goal of environmental intervention for chronic kidney disease (CKD) prevention.

## Introduction

Man-made and naturally occurring environmental exposures such as air pollution, heavy metals, and synthetic chemicals are increasingly associated with kidney impairment in adult and general populations.^1–3^ These environmental exposures can be inhaled, ingested, or absorbed through the skin, subsequently entering the bloodstream and reaching the kidneys.^3^ Kidneys are susceptible to environmental toxicants because they receive a large proportion of cardiac output compared to their size, have robust metabolic activity, and concentrate waste products for excretion, creating high local concentrations of toxicants immediately adjacent to kidney tubular cells.^4^

Many potentially hazardous environmental exposures begin well before adulthood, during critical periods of kidney development and maturation, including prenatal nephrogenesis and times of rapid growth and physiologic change in childhood and adolescence. However, detecting early kidney disease and injury in children is challenging, and therefore the kidney-related risks of environmental exposures in children have been understudied. As the global burden of CKD and its contributory cardiometabolic risk factors increases, there is mounting urgency to understand the modifiable relationship between environment and CKD risk and its roots in early life.^5–7^ Climate change, rising temperatures, and more frequent extreme weather events also intensify air and ground pollution and amplify this urgency.

Here, we review the current evidence on the potential contributions of air pollutants, heavy metals, and synthetic chemical exposures to 1) kidney impairment in children and 2) childhood cardiometabolic factors that increase their lifetime risk of CKD. We also present gaps in and future directions for research on environmental exposures and pediatric kidney outcomes.

We identified research aligned with the topic through December 2025, using searches in PubMed and Embase including key words such as ‘environment,’ ‘air pollution,’ ‘heavy metal,’ ‘chemical,’ ‘green space,’ and ‘environmental epidemiology,’ for studies relevant to kidney disease, hypertension, obesity/overweight and other metabolic conditions in children. Snowballing was used to identify additional relevant literature. We excluded case reports but considered all other study types for this narrative review. We prioritized studies that measured the specific outcomes of estimated glomerular filtration rate (eGFR), etiologic CKD diagnoses, urinary biomarkers, blood pressure, and body mass index in children (<18 years old). Secondarily, we prioritized review of similar studies in adult populations where pediatric data was lacking.

## Air pollution

Fine particulate matter < 2.5 μm in diameter (PM_2.5_) is a major contributor to air pollution and a threat to human health (Table 1). PM_2.5_ is a complex, geographically varied mixture of small particles, including organic carbon, elemental (black) carbon (BC), metal oxides, and inorganic ions like sulfate and nitrate, that can reach the bloodstream after inhalation.^8^ BC is an indicator of traffic-related pollution, and sulfates are indicators of coal combustion.^8^

**Table 1:** Summary of environmental toxicants and their potential effects on childhood kidney and cardiometabolic health.

Toxicants, *reference standards if available*	Kidney effects in children	Cardiometabolic effects in children	Major limitations of current studies
Air pollution			
**Particulate matter** < **2.5 μm (PM**_**2.5**_**)** *WHO recommended level* < *5 *μ*g/m*^*3*^ *(annual) or* < *15 *μ*g/m*^*3*^ *(24-hour); no direct clinical test*	Increased childhood exposure, especially above WHO annual guidelines, is associated with decreased eGFR in adolescence, increased incidence of CKD, and increased risk of glomerular disease progression. Increased prenatal exposure is associated with higher eGFR in adolescence.	Increased childhood exposure, especially above WHO annual guidelines, is associated with hypertension, overweight and obesity. Increased prenatal exposure, especially above WHO 24-hour guidelines, is associated with higher newborn SBP. Limited, possibly sex-specific, association between increased prenatal exposure and childhood overweight and obesity.	-Ecological designs that do not account for individual differences in physical activity, outdoor time, etc. -Most studies are limited to urban areas, leaving gaps in understanding of effects in rural and suburban populations. -Lack of consensus in outcome measurements for early kidney damage in children. For example, there is no standard definition of hyperfiltration and no widely validated battery of urinary protein biomarkers.
**Sulfate aerosols (component of PM**_**2.5**_**)**	Increased prenatal exposure is associated with increased incidence of and morbidity and mortality from CAKUT.	Limited associations with cardiometabolic health	
**Black carbon (component of PM**_**2.5**_**)**	Increased childhood exposure is associated with increased risk of glomerular disease progression.	Increased prenatal exposure is associated with higher newborn SBP.	
**Gaseous pollutants (e.g., NO**_**2**_**, SO**_**2**_**)** *WHO recommended NO*_*2*_ *level* < *10 *μ*g/m*^*2*^ *(annual) or* < *25 *μ*g/m*^*2*^ *(24-hour), SO*_*2*_ *level* < *40 *μ*g/m*^*2*^ *(24-hour); no direct clinical test*	Increased NO_2_ exposure, especially above WHO guidelines, is associated with increased incidence of pediatric CKD and lupus nephritis. Increased SO_2_ exposure is associated with CAKUT and weakly associated with reduced kidney function in sex-specific manner.	Limited associations with cardiometabolic health	
**Traffic pollution** (comprised of gases and particulate matter of varying sizes) *Broad grouping with varied guidelines*	Limited prenatal and childhood exposure associations with kidney impairment	Increased prenatal exposure is associated with higher total fat mass in childhood.	
Heavy metals			
**Arsenic** *WHO recommended level in drinking water* < *10 *μ*g/l; US CDC 24-hour urinary arsenic reference range* < *50 *μ*g/l*	Mixed associations between blood and spot urinary levels and kidney outcomes (eGFR and subclinical kidney injury markers)	Higher maternal blood levels prenatally associated with sex-specific effects on childhood adiposity	-Small sample sizes in heterogeneous populations -Surrogate exposure measures (e.g., single measurements of urinary heavy metals) of unclear significance -Confounding by unmeasured sociodemographic factors -Heavily cross-sectional designs that cannot rule out reverse causation and cannot measure cumulative exposure -Lack of consensus in outcome measurements for early kidney damage in children -Highly nuanced findings without replication
**Cadmium** *WHO recommended level in drinking water* < *3 *μ*g/l; Mayo Clinic urinary 24-hour cadmium reference range* < *3 *μ*g/g creatinine*	Mixed associations between blood and spot urinary levels and kidney outcomes (eGFR and subclinical kidney injury markers)	Limited and inconsistent associations between blood and spot urinary levels and childhood cardiometabolic health	
**Chromium** *WHO recommended level in drinking water* < *50 *μ*g/l; US CDC urinary 24-hour chromium reference range* < *10 *μ*g/l*	Limited positive associations between spot urinary levels and subclinical kidney injury markers	Limited and inconsistent associations between spot urinary levels and childhood cardiometabolic health	
**Copper** *WHO recommended level in drinking water* < *2000 *μ*g/l; Mayo Clinic serum copper reference range 40*–*180 *μ*g/dL in children*	Limited positive associations between spot urinary levels and subclinical kidney injury markers	Limited and inconsistent associations between spot urinary levels and childhood cardiometabolic health	
**Lead** *WHO recommended level in drinking water* < *10 *μ*g/l; US CDC blood lead level reference range* < *3.5 mg/dL*	Limited positive associations between low-level blood lead measurements in childhood and glomerular disease progression. Mixed associations between blood and spot urinary levels and other kidney outcomes (eGFR and markers of subclinical kidney injury).	BMI potentially modifies the relationship between blood lead levels and eGFR	
**Manganese** *WHO recommended level in drinking water* < *80 *μ*g/l; US CDC blood manganese reference range 4*–*15 *μ*g/l*	Higher maternal blood levels prenatally associated with lower risk of complicated CAKUT (study population IQR 12–20 μ*g/l)*	Potentially protective effect of higher maternal blood levels on childhood SBP	
**Vanadium** *No general community-based guidelines (only occupational thresholds); no widely reported reference range for direct clinical testing*	Limited positive association between spot urinary levels and abnormal eGFR and subclinical kidney injury markers	Limited and inconsistent associations between spot urinary levels and childhood cardiometabolic health	
Synthetic chemicals			
**Per- and polyfluoroalkyl substances (PFAS)** *US EPA drinking water MCL ranging from 4 to 10 ng/L depending on specific PFAS compound; no widely reported reference range for clinical testing*	Increased self-reported prenatal exposure associated with CAKUT in boys. Increased childhood plasma levels associated with lower eGFR in young adults.	Positive associations between prenatal and early childhood exposure measured in plasma and serum and childhood hypertension and obesity	-Sparse mechanistic data in humans -Confounding by unmeasured sociodemographic factors -Limited epidemiologic studies in vulnerable groups of children -Lack of consensus in outcome measurements for early kidney damage in children
**Insecticides (including organophosphates)** *Broad grouping with varied guidelines*	Increased self-reported prenatal exposure associated with CAKUT in girls. Increased childhood exposure measured via urine metabolites associated with subclinical markers of kidney injury but mixed association with eGFR in pediatric CKD prevalent population.	Limited associations with childhood cardiometabolic health	
**Organic solvents** *Broad grouping with varied guidelines*	Mixed associations between maternal exposures and CAKUT	Limited association with childhood cardiometabolic health	
**Herbicides (including glyphosate)** *Broad grouping with varied guidelines*	Mixed associations between urinary levels and childhood subclinical kidney injury markers	Limited association with childhood cardiometabolic health	
**Bisphenol A and phthalates** *US EPA drinking water di(2-ethylhexyl) phthalate MCL* < *0.0006 mg/l; varied clinical reference ranges*	Positive association between urinary levels and subclinical kidney injury markers in pediatric CKD prevalent population	Higher maternal urinary levels prenatally associated with childhood hypertension	
**Polycyclic aromatic hydrocarbons (PAH)** *US EPA drinking water PAH MCL* < *0.0002 mg/l*	No data available on association with pediatric kidney outcomes	Mixed associations between prenatal airborne exposure and childhood BMI	
**Organophosphate flame retardants (OPFRs)** *Broad grouping with varied guidelines*	No data available on association with pediatric kidney outcomes	Limited associations with childhood cardiometabolic health	

*BMI* body mass index, *CAKUT* congenital anomalies of the kidney and urinary tract, *CKD* chronic kidney disease, *eGFR* estimated glomerular filtration rate, *SBP* systolic blood pressure, *MCL* maximum contaminant limit, *US EPA* United States Environmental Protection Agency, *US CDC* United States Centers for Disease Control, *WHO* World Health Organization, Sources: CDC ASTDR Toxic Substances Portal & Clinical Assessment –Laboratory Tests (2023), WHO Global Air Quality Guidelines (2021) & Guidelines for Drinking-water Quality (2022), Mayo Clinic Pediatric Test Catalog (2025), EPA national primary drinking water regulations (2026).

In urban areas, major PM_2.5_ contributors include diesel and other vehicular exhaust, industrial emissions, and residential heating. In rural areas, major contributors are agricultural activities, biomass burning, and wind-blown dust.^9^ Wildfires, increasing in frequency and severity globally, also emit PM_2.5_ with high contents of toxic organic compounds.^10^

In addition to particulate matter, gases like nitrogen dioxide (NO_2_) and sulfur dioxide (SO_2_) are also produced by combustion of fossil fuels, industrial and agricultural activities, and landscape fires. Gaseous pollutants ultimately contribute to PM_2.5_ through formation of secondary particulate matter but also have independent impacts on human health.^11^

In animal models, PM_2.5_ stimulates oxidant stress and cytokine response that can contribute to end organ damage. Reactive nitrogen species, such as those derived from NO_2,_ also interact with cellular components and affect cell signaling.^12^ Compared to mice exposed to filtered air, mice exposed to urban PM_2.5_ experience reduced glomerular filtration, impaired urine concentration ability, and extensive tubular damage in the setting of ischemia-reperfusion kidney injury, which is used to create acute kidney injury (AKI) in mice. This suggests that PM_2.5_ exposure may heighten susceptibility to CKD in individuals experiencing AKI.^13^ Additionally, maternal PM_2.5_ exposure during nephrogenesis in rat pups was found to increase glomerular, tubulointerstitial, and cortical kidney damage in the pups.^14^

Studies in human populations with heightened exposure to air pollution are needed to confirm mechanistic findings from animal models. Notably, in healthy individuals, inhaled inert gold nanoparticles—a model for PM_2.5_ exposure—are detected in urine within 15 min of exposure and still detectable three months after exposure, highlighting the potential cumulative and direct impact of PM_2.5_ on the kidney.^15^

Children exposed to air pollution through wildfire smoke represent an important population to understand and protect. Children often have heightened exposure to air pollution due to their small airways, developing immunity, and predilection for outdoor time, and wildfire smoke is particularly toxic due to its high content of carbonaceous and polar organic compounds.^10,16^ Wildfire smoke has systemic impacts on human health through inflammation, oxidative stress, weakened DNA repair mechanisms, and epigenetic modulation of gene promotors.^16^ However, a recent systematic review assessing the impact of wildfire smoke on children revealed that none of the 16 identified studies measured kidney-related outcomes, despite the well-established concept of lung-kidney cross-talk.^10,17^

### Pediatric CKD development and progression

Although there is limited mechanistic knowledge, air pollution is epidemiologically linked to congenital anomalies of kidney and urinary tract (CAKUT) and to pediatric glomerular disease, which comprise the two most common causes of CKD in children.^8,18^ A global ecological study evaluating national-level air pollution emission data from 1990 to 2021 found that SO_2_ is associated with incidence of and morbidity and mortality from urogenital congenital anomalies.^19^ In two large observational glomerular disease cohorts in the United States that include children, census tract-level estimates showed that participants with doubling of baseline PM_2.5_ had 55% increased risk of disease progression. Further evaluation of specific components of PM_2.5_ demonstrated that doubling of the BC component had 43% increased risk of disease progression, but there was no association between sulfates and disease progression.^8^ In a multicenter longitudinal study of children and adults with lupus in China, short-term exposures to PM_2.5_ and NO_2_, but not SO_2,_ were identified as risk factors for lupus nephritis within one month after increased exposures.^20^

While intriguing, these studies are limited by at least partially ecologic designs. With ecologic exposure measurements, all patients are assigned the exposure level of the subgroup, which may not be correct, and other factors that are correlated within similar environments may be confounding factors.^21^

### Pediatric kidney impairment

Outside of specific CKD etiologies, air pollution has been associated more generally with decreased childhood kidney function in some studies. One study followed 427 mother-child dyads throughout pregnancy and early childhood in Mexico City, estimating daily PM_2.5_ exposure at each dyad’s residence and measuring childhood estimated glomerular filtration rate (eGFR).^22^ eGFR is measured using serum creatinine or serum cystatin C and is the most widely accepted measure of kidney function. Early pregnancy PM_2.5_ exposure was associated with higher eGFR in preadolescence. This could suggest glomerular hyperfiltration as the first sign of chronic injury. However, this conclusion is limited because there is no standardly accepted cut point between normal eGFR and hyperfiltering eGFR in children.^23^ In contrast, PM_2.5_ exposures in the first 14 months after birth were associated with decreased preadolescent eGFR.^22^ Similarly, in a large longitudinal study in Taiwan and Hong Kong, each 10 μg/m^3^ increase in PM_2.5_ was associated with 53% greater risk of incident CKD in young people between 2 and 25 years old.^24^

With regard to gaseous pollutants, long-term exposure to NO_2_ is associated with pediatric CKD. Each 10 μg/m^3^ increase in NO_2,_ measured over a 2-year period using satellite-based spatiotemporal regression models, is associated with 7% higher risk of incident CKD in children living in Taiwan.^24^ SO_2_ exposure has not been as well studied but is weakly associated with reduced kidney function in a sex-dependent manner.^25^

### Childhood cardiometabolic risk factors for CKD

Air pollution also impacts risk for hypertension and adiposity, which are two main cardiometabolic risk factors for CKD.^26,27^ Children with hypertension and increased adiposity are at higher risk for CKD in young adulthood, which is associated with faster progression to end-stage kidney disease (ESKD) compared to CKD in older adults.^6,28,29^ Multiple meta-analyses have concluded that short- and long-term PM_2.5_ exposures in childhood are associated with pediatric hypertension.^30–32^ One study found that higher mean PM_2.5_ and BC exposures in the third trimester are associated with higher systolic blood pressure (SBP) in newborns, although the significance of higher SBP still within the normal range is unclear.^33^ The study did not report whether any of the infants had elevated SBP based on percentile.

Many studies have found that exposure to PM_2.5_ and its constituents in childhood is associated with overweight and obesity in children, with a pooled analysis demonstrating significantly increased risk at PM_2.5_ levels above 35 μg/m^3^.^34^ A cohort study of children in Boston found that prenatal and postnatal PM_2.5_ exposure beyond a daily median of 10.5 μg/m^3^ demonstrates a dose-response relationship with later childhood overweight and obesity.^35^

Prenatal exposure to traffic pollution, of which PM_2.5_ and gases are two of several components, has also been associated with overweight and higher total fat mass in mid-childhood.^36,37^ However, associations specifically between prenatal exposure to PM_2.5_ and childhood metabolic risk factors are limited and possibly sex-specific.^36–38^

PM_2.5_ is postulated to impair vascular function by triggering systemic inflammation and oxidative stress.^39,40^ Additionally, rodent models consistently show PM_2.5_ -induced inflammatory responses in adipose tissue.^35,41,42^ Unsurprisingly, these postulated mechanisms are similar to those thought to contribute directly to kidney function decline.^32^

## Heavy metals

Heavy metals are both naturally found in and common contaminants of water, food, and soil, released by human activities like mining and industrial processes.^43^ Agricultural fertilizers and sewage also add metals to soil, and agricultural runoff carries them into nearby water systems.^44^ From 2010 to 2017, concentrations of heavy metals such as cadmium, lead, and mercury in North American rivers and lakes increased to levels more than nine times higher than World Health Organization (WHO)-recommended thresholds.^45^

Children can have intensified heavy metal exposures through ingestion of contaminated food or water and hand-to-mouth ingestion after contact with paint and ground matter. Heavy metals can cross the placenta and into breast milk as well as contaminate infant formula.^4^ Infants may be disproportionately affected by heavy metal contamination of fluids due to higher consumption of liquid volume per unit of body weight than adults.^46^ Adolescents working in certain industrial occupations are also susceptible to the effects of heavy metal exposure, especially if they are not given proper education on working conditions and hygiene practices.^47^

Single heavy metals as well as metal mixtures are associated with CKD risk (Table 1).^1^ Animal studies suggest that heavy metals disturb kidney hemodynamics, leading to elevated levels of reactive oxygen species in kidney tissues and resulting in varying degrees of chronic kidney tubular injury and fibrosis.^48^ In zebrafish *Danio rerio*, heavy metal mixtures were found to alter gene expression markers of kidney development and kidney injury.^49^ Heavy metals and other drinking water contaminants have also been implicated in mitochondrial dysfunction, which is another potential mechanistic driver of CKD.^49^

### Pediatric CKD development and progression

Literature evaluating the association of heavy metals with incidence and progression of specific etiologies of pediatric CKD is scarce. A cohort study in Japan investigated the association between heavy metals and CAKUT, finding that higher maternal blood manganese concentration was associated with lower risk of complicated CAKUT.^50^ No associations were found between maternal blood levels of lead, cadmium, and mercury and CAKUT. In a cross-sectional analysis of a CKD prevalent pediatric cohort, no overall association was seen between blood lead levels and directly measured GFR.^51^

### Pediatric kidney impairment in cross-sectional studies

Several cross-sectional analyses have demonstrated a relationship between higher exposures to heavy metals and lower eGFR.^1^ For example, in rural Bangladesh, each doubling of urine cadmium level was associated with an eGFR decrease of 4.29 ml/min/1.73 m^2^ in 9-year-old children.^52^ However, reduction in eGFR is a relatively late marker of kidney injury, occurring after substantial damage to kidney tissue.

Other cross-sectional studies have looked at earlier subclinical but less validated markers of kidney injury. In children living in Mexico, increased urinary levels of arsenic, cadmium, copper, and vanadium are associated with altered expression of urinary cystatin C, alpha-1 microglobulin, neutrophil gelatinase-associated lipocalin, and kidney injury marker-1 (KIM-1), which are all protein biomarkers of early kidney damage.^53–55^ Urinary cadmium even at low levels is associated with increased levels of subclinical kidney damage biomarkers.^56^ Another study in Mexico also found that urinary KIM-1 demonstrated a dose-dependent relationship with urinary chromium and was associated with the upper tertile of urinary arsenic, suggesting that urinary KIM-1 could serve as a sensitive biomarker for both kidney and environmental risk assessment in children.^57^

Nevertheless, cross-sectional studies evaluating heavy metal levels and kidney outcomes should be approached with caution because the kidney is the main organ for excreting heavy metals. Studies may present heavy metals as the exposures and kidney dysfunction as the outcome, but reverse causation is difficult to rule out in cross-sectional measurements. For example, studies utilizing the National Health and Nutrition Examination Survey have found that increased levels of single heavy metals as well as metal mixtures measured in urine are associated with higher eGFR, but—due to the cross-sectional nature and lack of consensus definition for hyperfiltration—we cannot say whether this represents kidney hyperfiltration resulting from heavy metal toxicity or expected toxicant excretion in the setting of high normal eGFR.^55,58–60^

### Pediatric kidney impairment in longitudinal studies

Few studies have evaluated the relationship between longitudinal, cumulative exposure to heavy metals and kidney outcomes later in childhood. In a cohort of children living in Mexico City, cumulative dietary cadmium over five study visits from birth to nine years old was not associated with eGFR, differing from the strong association detected in the previously mentioned cross-sectional study from rural Bangladesh.^52,61^ Longitudinal analyses of second-trimester heavy metal exposures measured in maternal blood and urine and biomarkers of subclinical kidney injury measured in their children between 8 and 12 years old have similarly shown mixed results.^62^

**Fig. 1:**
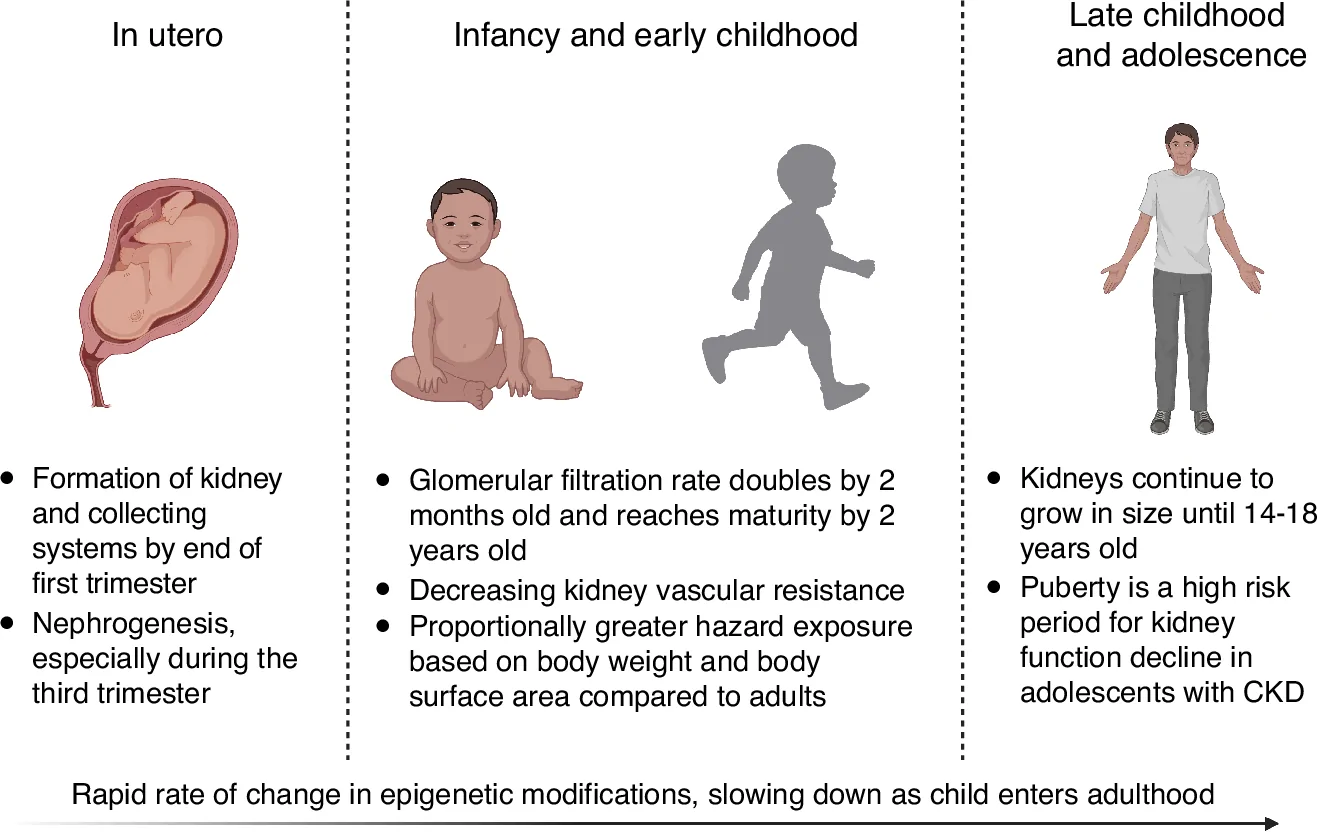
Critical windows in childhood kidney development that may result in differential effects of environmental exposures. CKD chronic kidney disease. Created with BioRender.com.

Instead of measuring heavy metals in urine, one longitudinal study measured prenatal metal exposure based on metal content in naturally shed deciduous teeth, using laser ablation-inductively coupled plasma-mass spectrometry (LA-ICP-MS) to identify the “neonatal line,” which is a histological feature formed at birth, and daily growth markings.^63^ The study reported that lead exposure in the third trimester is linked to higher preadolescent eGFR, whereas exposure to a metal mixture of arsenic, cadmium, lead, chromium, and lithium between 8–17 weeks’ gestation is associated with decreased preadolescent eGFR.^63^ However, effect sizes were not reported, so it is unclear whether the overall increase or decrease in eGFR is clinically meaningful. Still, these findings, coupled with the previously discussed longitudinal study of PM_2.5_ effect in early life versus later childhood on eGFR, could suggest critical windows of childhood development during which the kidneys are differentially susceptible to environmental exposures (Fig. 1).^22,63^

More so than other potential toxicants discussed in this review, heavy metals have been studied in a variety of pediatric populations, including those living in rural and urban areas and in countries with different degrees of development. From these studies, general levels of heavy metal exposures for children around the world can be gleaned. However, greater public health investment in pediatric longitudinal research studies is necessary to determine the levels at which heavy metals and mixtures become toxic to the kidneys and whether acceptable heavy metal levels and screening recommendations should be reevaluated.

### Childhood cardiometabolic risk factors for CKD

Exposure to heavy metals such as lead, arsenic, and cadmium contributes to cardiometabolic risk factors in adults through endothelial dysfunction and oxidative stress.^64,65^ However, studies in children have yielded mixed and inconsistent results.^52,66,67^

In the Boston Birth Cohort, maternal heavy metal levels, including lead, cadmium, and manganese, were measured perinatally in blood samples and were not associated with elevated SBP later in childhood.^68^ Similar to the previously discussed potential protective effect of manganese on CAKUT risk, there was a protective association of manganese on childhood SBP for those born to mothers who had higher cadmium levels.^68^ Manganese is postulated to exert a protective effect through activation of the antioxidant enzyme manganese superoxide dismutase.^69^

Body mass index (BMI) has been found to modify the relationship between blood lead levels and eGFR. One study reported that higher blood lead levels are associated with decreased eGFR in overweight preadolescents only.^70^ Additionally, maternal blood arsenic levels measured in the third trimester—but not levels measured earlier in pregnancy or later in childhood—are weakly associated with increased adiposity later in childhood for females but not males, again highlighting susceptibility that varies according to developmental windows, perhaps with sexual dimorphism.^71^ These findings may also represent chance findings due to decreased power as subgroups and variable interactions increase and require external validation in other populations.

## Synthetic chemicals

Globally, more than 2.3 billion tons of synthetic chemicals are produced annually, including millions of new chemicals used in industry, agriculture, and the household.^72^ Humans encounter these chemical pollutants in water, food, and through dermal contact.^3^ The Environmental Protection Agency (EPA) estimates that only 1% of chemicals present in household products have undergone stringent safety testing.^72,73^ However, several modern chemicals—such as phthalates, phenols, per- and polyfluoroalkyl substances (PFAS), organophosphates, and glyphosates—are now linked to adverse effects on human health (Table 1).^26,72,74^ Animal models demonstrate that in utero and early childhood exposure to these chemicals could increase lifetime risk for kidney and cardiometabolic disease through heightened oxidative stress, aberrant renin-angiotensin system, dysregulation of the aryl hydrocarbon receptor, and lower nephron number.^26^

### Pediatric CKD development and progression

Examination of the relationship between chemical toxicants and various etiologies and progression of pediatric CKD is limited. In the Japan Environment and Children’s Study, mothers self-reported use of household products such as organic solvents, waterproof sprays, insecticides, and pesticides during pregnancy, and the measured outcome was CAKUT diagnosed by the first year after birth.^75^ Prenatal use of waterproof spray, which frequently contains PFAS due to their non-stick properties, was associated with 28% higher odds of urological anomalies in boys, and prenatal use of insecticides was associated with 48% higher odds of urological anomalies in girls. There were especially strong associations between prenatal use of waterproof spray and vesicoureteral reflux in boys and prenatal use of insecticides and hydronephrosis in girls. No association was detected between maternal exposure to organic solvents (used in paints, glues, and cleaning products) and CAKUT.^75^ However, in a European case-control study of maternal occupational exposure in the first trimester, organic solvents/alkylphenolic compounds were associated with 41% higher odds of CAKUT.^76^

In a pediatric CKD prevalent population, exposure to organophosphates, used in agriculture as insecticides, was associated with subclinical markers of kidney injury and oxidant stress throughout a 10 year follow up period.^77^ Bisphenol A and phthalates, both plasticizers, have also been studied in relation to pediatric CKD with mixed findings.^78,79^ Neither has been consistently associated with eGFR decline, but, similar to organophosphates, both are associated with markers of kidney tubular injury and oxidative stress.^78,80^ Thus, chemical toxicants may increase the risk of pediatric CKD development and progression, but more investigation is needed.

### Pediatric kidney impairment

PFAS burden score, developed to estimate exposure to these chemical mixtures, is associated with lower eGFR in young adults of the US-based Children’s Health Study.^81^ Additionally, synthetic agrochemicals are probable contributors to the pathogenesis of chronic kidney disease of unknown etiology (CKDu), also known as chronic interstitial nephritis in agricultural communities (CINAC), which is an epidemic of kidney disease in young adults living and working in agricultural hotspots around the globe.^82^ CKDu-related kidney injury likely begins in childhood.^83^ In Mexico, which has CKDu-endemic hotspots, higher levels of urinary KIM-1 were seen in children with levels of glyphosate (a popular synthetic herbicide and weed killer) in the top quartile.^84^ However, a study of children in the US did not identify any significant associations between urinary glyphosate levels and urinary biomarkers of kidney injury, including KIM-1.^85^ Overall, the literature regarding the relationship between agrochemicals and kidney health in children remains sparse.

### Childhood cardiometabolic risk factors for CKD

PFAS exposures prenatally and in early childhood are positively associated with hypertension and obesity in adolescence.^86,87^ Additionally, prenatal exposure to monobutyl phthalate, a phthalate ester, is associated with increased odds of childhood hypertension.^88^ Phthalate metabolites measured later in childhood have not demonstrated the same link to childhood hypertension, further highlighting that the prenatal period is a critical window of childhood development during which cardiac and kidney health may be especially susceptible to environmental exposures.^63,89^

Other chemicals such as polycyclic aromatic hydrocarbons (PAH), commonly encountered as byproducts of incomplete combustion of asphalt, coal, and tobacco, and organophosphate flame retardants (OPFRs) can bind to the fine particles that make up PM_2.5_.^90^ Prenatal exposures to airborne PAH have been associated with higher childhood BMI Z-score at 4 years old but not at 11 years old.^91^ OPFRs are associated with nephrotoxicity in adults, but their effect in children is not well understood.^92^

## Environment as protection

The environment can be modified or preserved to protect childhood kidney and cardiometabolic health.^93^ While interventions such as decreasing air pollution may require complicated policy and regulatory negotiations, other potentially protective interventions, such as increasing green space, are more tenable at a community level and through grassroots efforts.^94^ Green spaces immobilize, absorb, and filter ambient particulate matter, heavy metals, and synthetic chemicals—in theory, significantly decreasing human exposure to these toxicants.^95,96^

Evidence of the effects of green spaces on childhood cardiometabolic health is inconsistent and heavily cross-sectional.^97–99^ Of mention, in a large cohort study of schoolchildren in China, green space was associated with decreased risk of elevated blood pressure over three years of follow-up.^100^ Several cross-sectional studies have found that children living in greener neighborhoods have lower BMI, but longitudinal studies have not yielded the same statistically significant results.^97,101,102^

With regard to kidney disease, a study of more than 600,000 adults in Taiwan found that proximity to green spaces was associated with lower hazards of ESKD in a CKD prevalent population.^103^ On the other hand, no association was seen between greenness and adult CKD in a cross-sectional Taiwanese sample.^104^ Both adjusted for sociodemographic variables. These studies are challenging to interpret because it is not clear if these associations are related to detoxifying effects of the green environment or to correlated factors like space for physical activity or other socioeconomic factors. To our knowledge, no studies have evaluated the association between environmental greenness and longitudinal risk of pediatric CKD onset or progression.

Importantly, the built environment can encourage health behaviors that are associated with better kidney and cardiometabolic health. Safe and walkable neighborhoods promote physical activity, which has protective effects on the cardiometabolic health of children exposed to high levels of environmental stress.^105,106^ Another important aspect of the built environment is access to fresh and varied food sources. Food insecurity is associated with an increased likelihood of high blood pressure.^107^ Cross-sectional studies have also found that food insecurity is associated with 35–46% higher odds of CKD.^108,109^ In contrast, food assistance for children with CKD may mitigate kidney disease progression.^110^

## Study power challenges and possible solutions

**Fig. 2:**
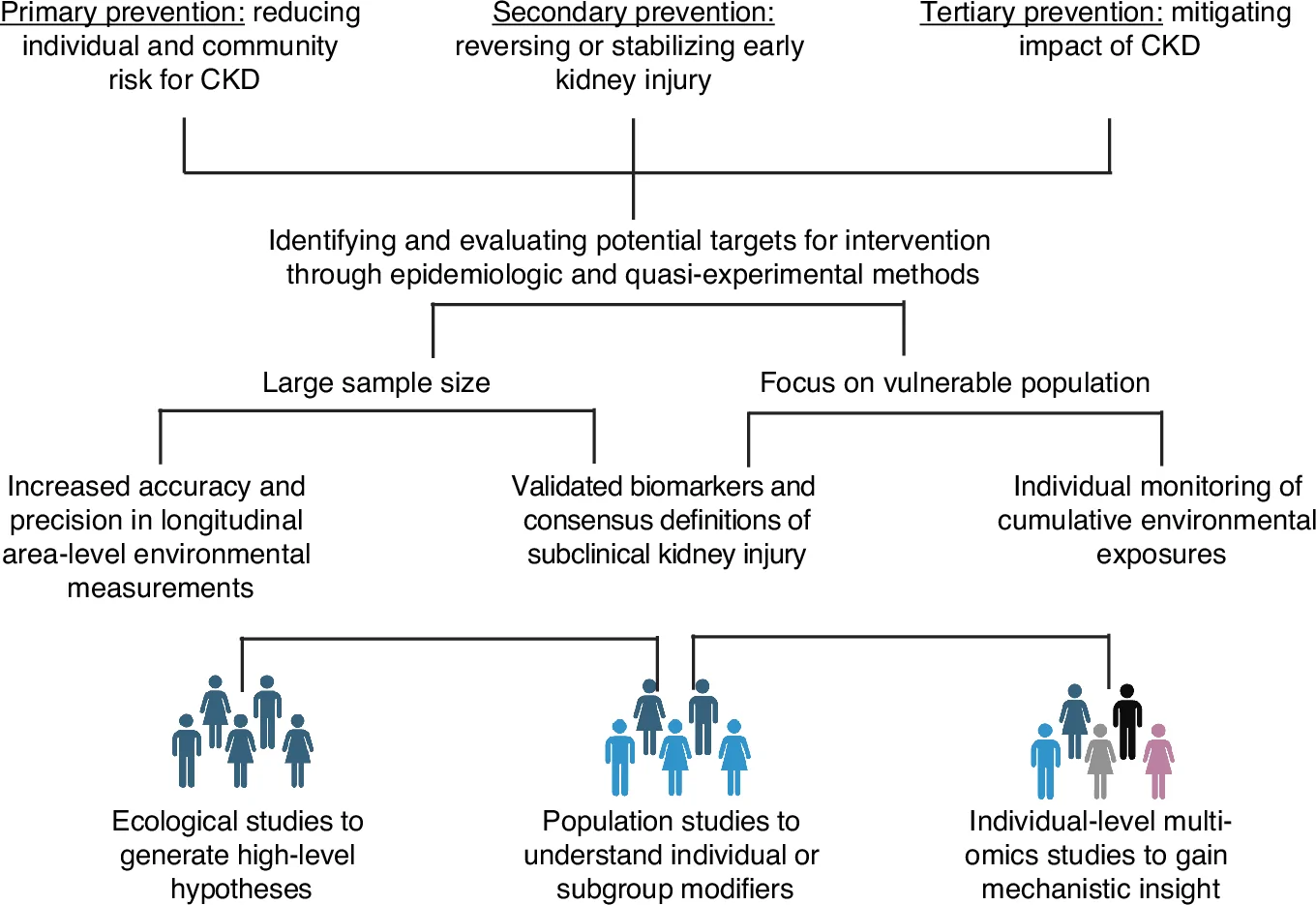
Potential applications of environmental health research to chronic kidney disease (CKD) prevention. Both large studies that clarify which toxicants contribute most to kidney disease and smaller precision health studies that describe environmentally related kidney injury in at-risk individuals are key to informing public health and individual-level prevention strategies. Colors are used to indicate measured exposure heterogeneity within populations. Created with BioRender.com.

In reality, humans usually experience environmental toxicants not as isolated exposures but instead as complex, dynamic mixtures of multiple metals, chemicals, and compounds.^111^ Metal and glyphosate-metal mixtures have demonstrated highly mixture-specific effects on kidney development.^49^ Moreover, there are millions of new chemicals and potential toxic substances released into the environment annually.^72^ The complexity of manifold, time-varying exposure combinations that influence kidney outcomes creates challenges for study design, highlighting the importance of thoughtfully powered studies to detect associations of clinical and public health relevance (Fig. 2).^112^ The following features are particularly important to consider in approaches to environment and kidney health research in order to overcome prior limitations.

### Sampling frame

Others have pointed to small sample sizes as limitations of previous studies.^113^ While larger sample sizes provide more power for assessing differences in heterogeneous populations, yet another approach is to study a more specific population.^112^ Nationally-representative studies can help identify which environmental exposures may be relevant to the kidney health of the largest number of people. However, environmental exposures often affect populations unevenly, and usually there is a smaller subgroup that is disproportionately affected.^112^ Thus, exposures of potential concern identified in larger heterogeneous populations should be tested at the community- and neighborhood- levels (i.e., in a population with a narrower distribution of the exposure variable) to increase sensitivity for detecting a relationship between the exposure and kidney health.^3,114,115^

For example, the CKDu in Agricultural Communities (CURE) Research Consortium is focusing on adults in CKDu-endemic areas to limit confounding and increase power to detect relationships between environmental exposures and kidney disease.^116^ The same should be done for children living in CKDu-endemic areas.^117^ Other vulnerable populations include children living in areas with water scarcity, groundwater well contamination, limited resources for maintenance and testing of private wells, and exposure to temperature extremes.^118,119^ More broadly, minority and socioeconomically disadvantaged communities disproportionately bear exposure to environmental toxicants, including air and chemical pollution, and associated burden of disease.^8,120^ Engaging these highly exposed local populations will increase study power as well as sharpen environmental health research as an advocacy tool.

### Accurate and precise environmental measurements

Accuracy is the correctness with which a measurement reflects the true value of an assessed exposure. Precision is the degree of variation in repeated exposure measurements.^121^ High degrees of accuracy and precision in environmental measurements is challenging because of their seasonal variance, accumulation over time, and complex mixtures. Despite rapid developments in analytical chemistry technologies, operationalizing the “exposome”—the totality of a person’s exposures, including prenatally—remains elusive.^115^

Current environmental sampling strategies have several limitations. As shown in Table 1, many national and intergovernmental organizations have guidelines for acceptable levels of various toxicants in environmental sources, but most studies measure toxicant levels in blood or urine tests.^117^ Many of these tests do not have validated reference ranges, only capture recent exposures, and are frequently conducted as spot tests instead of gold-standard 24 h measurements. Moreover, even with established clinical reference ranges for heavy metals, there is growing evidence that low-level (i.e., below the clinical reference range) but persistent lifetime exposure to environmental toxicants poses substantial risk to human health.^122^

Self-reported environmental exposures are also common but subject to inaccuracy due to recall bias.^123^ Blood heavy metal levels may also inaccurately portray the relationship between exposure and kidney outcomes, as higher blood heavy metal levels may be a consequence rather than a cause of decreased kidney function.^4^ Precision in measuring urinary heavy metal levels has been achieved through adjustment for urinary concentration by urine creatinine (either in statistical modeling or with simple ratios), urinary osmolality, or urine flow rates, but these varied approaches provide discordant results that cannot be readily compared across studies.^4,113,124^ Additionally, despite a proliferation of geospatial data, evaluation of how individuals interact with their geospatial environment is often lacking, predisposing to ecological fallacy and important but unmeasured confounders.^101^

Individual monitors for a range of exposures from air pollution to synthetic chemicals are becoming more widely available and will be instrumental in overcoming the limitations of ecologic exposure methods.^115^ As previously mentioned, one study used deciduous teeth that allow for increased precision in determining an individual’s historical and cumulative exposure.^1,63^ Prioritizing longitudinal data that includes multiple timepoints of environmental measurements at different levels (i.e., individual exposure and community exposure), with the goal of greater accuracy and precision, increases study power by reducing random error, unexplained variance, and overall bias.^125^

### Validated subclinical outcome measurements

Abnormal serum creatinine and cystatin C, used to measure eGFR, are late markers of CKD. Urine albumin-to-creatinine ratio (UACR) is also a relatively late marker and, while frequently employed as an outcome in adult CKD studies, it is not as widely measured in pediatric community-based studies. UACR is limited in pediatric evaluation due to the need to collect first morning urine samples to rule out benign orthostatic albuminuria and because pediatric CKD is more commonly non-albuminuric.^18^

eGFR and UACR as outcome measurements are useful in characterizing the effects of environmental exposures on CKD progression, but subclinical, early kidney injury markers are currently lacking and critically needed for detecting injury when it may still be reversible.^126–128^ Validated and unified subclinical kidney outcome markers are also needed to strengthen conclusions across studies.^129^ Candidates for a standardized battery of biomarkers for early detection of kidney injury include protein biomarkers, such as KIM-1 and NGAL, that can be traced to known pathophysiological processes involved in CKD progression and non-coding RNAs, such as microRNAs (miRNAs) and long non-coding RNAs (lncRNAs), that are involved in tissue-specific gene regulation.^126,130,131^ Biomarker studies could also uncover distinct mechanisms and molecular phenotypes of environmental kidney injury.

The epigenome may provide another reversible early marker of kidney injury.^132^ Accelerated epigenetic age in early life has been associated with poorer cardiometabolic health later in childhood, and epigenetic modifications are implicated in kidney disease development.^115,133,134^ Many of the postulated mechanisms of environmentally-driven kidney damage, such as mitochondrial dysfunction and increased reactive oxygen species leading to oxidative stress and fibrosis, are hallmarks of senescence and aging. Development of a kidney-specific epigenetic age acceleration clock that can be measured through blood samples would be helpful in discerning environmental drivers of kidney disease, as well as providing a potentially reversible target in longitudinal studies measuring hazard reduction.^115,135,136^

### Evaluating environmental interventions

The growing and promising capabilities of environment-wide association studies coupled with multi-omic analyses to identify markers of exposure and predictors of kidney health are frequently discussed.^3^ As targets for environmental intervention are identified, quasi-experimental study designs may be useful in elucidating the impact of environmental hazard reduction. Quasi-experimental designs including difference-in-differences (DID) and instrumental variable approaches would improve causal inference, overcome limitations of existing ecologic and cross-sectional studies, and aid in approximating effects of public health interventions and policies.

DID estimates the intervention effect by comparing the change in outcomes for the experimental group to a control group with a trend that approximates what would happen in the experimental group without the intervention, thereby accounting for neighborhood and individual factors that are time invariant or slowly varying.^137,138^ Instrumental variables can be challenging to identify, but if available, can overcome confounding by isolating the effect of an exposure using only the variation induced by a ‘randomizer’, the instrument. These quasi-experimental designs should be considered with the goal of providing internally and externally validated conclusions on the relationship between environmental hazard reduction and changes in kidney health.^139–141^

### Community action

While research strategies continue to be optimized, communities can take immediate steps to protect their health by reducing exposures to environmental toxicants. Most practically, clinicians and community members can increase awareness of two existing toxicant monitoring tools: local air quality monitoring and lead screening in children. Following the local air quality index allows individuals to be alerted when air pollution is at levels especially harmful to human health, allowing them to modify their outdoor time during those periods.^142^ Lead screening should be considered for all children, although insurance coverage may vary. Even low-level environmental lead exposure can contribute to progressive decline in eGFR.^143,144^

Beyond these tools, there are opportunities for child health experts to learn more about environmental exposures and childhood health. The World Health Organization has a self-paced “Environment, climate change, and health” course that includes modules on air pollution and chemical safety, and certain national organizations, such as Pediatric Environmental Health Safety Units (PEHSU) in the US, provide community-level education on environmental exposures.^145,146^ A comprehensive list of resources for global environmental health education to protect the health of children can be found in the American Academy of Pediatrics’ “Green Book” and recent technical report on “Environmental Issues in Global Pediatric Health.”^147,148^ Equipped with these resources, child health experts can incorporate environmental determinants of health into medical education and routine care, better answer questions about environmental causation that arise during clinic visits, and play key roles in advocating for policy, research, and infrastructure that promotes greener, healthier communities.

## Summary and future directions

**Fig. 3:**
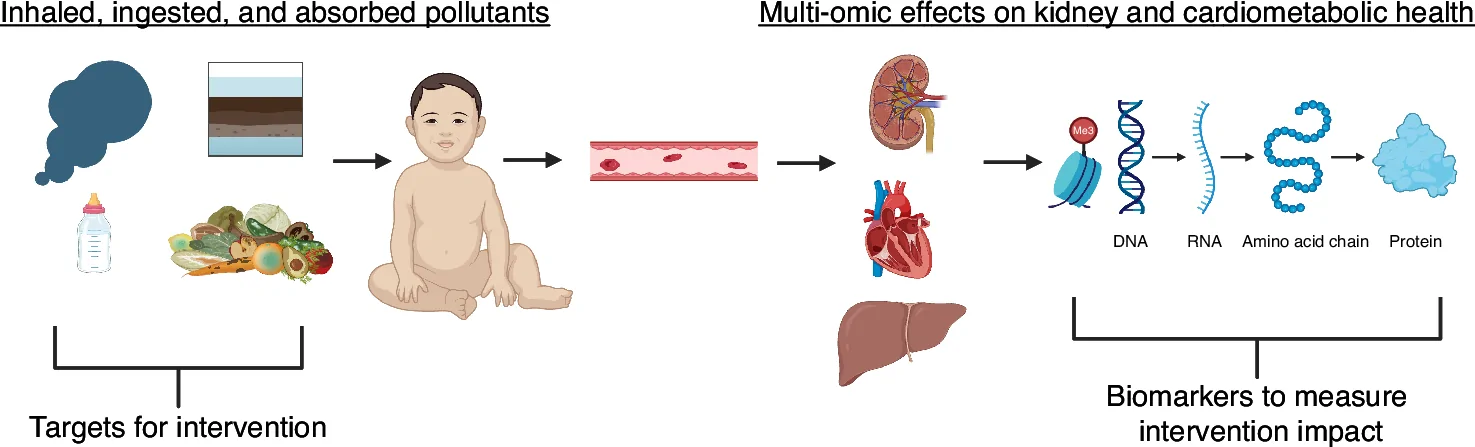
Summary of environmental exposures as potentially modifiable risk factors for kidney disease. Environmental exposures that are inhaled, ingested, or absorbed can cross into the bloodstream and influence early life kidney health and development. Some exposures may directly contribute to kidney injury, while others may indirectly contribute by driving cardiometabolic risk factors for kidney injury such as hypertension and obesity. A multi-omics approach to understanding mechanisms and molecular phenotypes of environmentally-related kidney injury may be key to early detection and prevention. Created with BioRender.com.

Environmental exposures are emerging as important drivers of pediatric kidney disease and pediatric cardiometabolic risk factors for CKD (Fig. 3). To date, investigations of relationships between manifold environmental exposures and kidney health outcomes have produced mixed results. Practical steps for clarifying these mixed associations include (1) combining epidemiologic, quasi-experimental, and mechanistic studies to interrogate effect sizes in especially vulnerable pediatric populations, (2) prioritizing prospective studies to assess lasting impacts of environmental exposures during critical windows for kidney development, and (3) standardizing environmental exposure and early kidney impairment measurements to facilitate cross-study comparisons. These steps move toward the overarching goal of identifying environmental targets for kidney and cardiometabolic disease prevention at the individual and community levels, starting in childhood.
